# Features on MDCT That Predict Surgery in Patients with Adhesive-Related Small Bowel Obstruction

**DOI:** 10.1371/journal.pone.0089804

**Published:** 2014-02-24

**Authors:** Wei-Chou Chang, Kai-Hsiung Ko, Chun-Shu Lin, Hsian-He Hsu, Shih-Hung Tsai, Hsiu-Lung Fan, Ho-Jui Tung, Guo-Shu Huang, Ran-Chou Chen

**Affiliations:** 1 Department of Radiology, Tri-Service General Hospital, National Defense Medical Center, Taipei, Taiwan; 2 Department of Biomedical Imaging and Radiological Sciences, National Yang-Ming University, Taipei, Taiwan; 3 Department of Radiation Oncology, Tri-Service General Hospital, National Defense Medical Center, Taipei, Taiwan; 4 Department of Emergency Medicine, Tri-Service General Hospital, National Defense Medical Center, Taipei, Taiwan; 5 Department of Surgery, Tri-Service General Hospital, National Defense Medical Center, Taipei, Taiwan; 6 Department of Healthcare Administration, Asia University, Taichung, Taiwan; 7 Department of Radiology, Taipei City Hospital, Taipei, Taiwan; Banner Alzheimer's Institute, United States of America

## Abstract

**Purpose:**

The purpose of this study was to determine the contribution of multidetector-row computed tomography (MDCT) in the management of adhesion-related small bowel obstruction (SBO) and to identify its predictive value for surgery.

**Methods:**

We conducted a retrospective review of 151 patients over a 5-year period with the diagnosis of SBO caused by adhesion. These patients were divided into two groups: surgery (n =  63) and observation group (n =  88). Two radiologists blinded to the outcome of the patients evaluated MDCT images retrospectively, recording the bowel diameter, bowel wall thickness, degree of obstruction, air-fluid level, mesenteric fatty stranding, transitional zone, intraperitoneal fluid, close loop, whirl sign, and faeces sign. Statistical analyses were performed using univariate and multivariable analyses.

**Results:**

Multivariable analysis showed that MDCT demonstrated presence of intraperitoneal fluid (Odds ratio, OR, 4.38), high-grade or complete obstruction (OR, 3.19) and mesenteric fatty stranding (OR, 2.81), and absence of faeces sign (OR, 2.11) were the most significant predictors**.** When all of the four criteria were used in combination, high sensitivity of 98.4% and specificity of 90.9% were achieved for the prediction for surgery.

**Conclusion:**

MDCT is useful to evaluate adhesion-related SBO and to predict accurately patients who require surgery. Use of the four MDCT features in combination is highly suggestive of the need for early surgical intervention.

## Introduction

Small bowel obstruction (SBO) is a common clinical entity with signs and symptoms mimicking other aetiologies of acute abdomen. Adhesions are the leading cause of blockage in the small intestines, causing at least 60% of all cases of SBO and accounting for 20% of all acute surgical admissions [1]–[4]. An adhesion-induced bowel obstruction is a dynamic and ever-changing process that can progressively evolve into a devastating condition with ischaemia and/or strangulation, or can resolve spontaneously. In general, it is accepted that patients with adhesion-related SBO should be treated conservatively when obstruction is first detected [3], [5]. If SBO is complicated with definite ischaemia, strangulation or perforation and these problems can be identified easily, then there is no question that a patient should receive surgery [6], [7]. However, in clinical practice, it is sometimes difficult to decide the appropriate timing for surgery, if only because of the variation in clinical symptoms that cannot accurately predict the development of strangulation or other severe complications. Therefore, radiological examinations play an important role in deciding the severity of the disease and progression of the obstruction [2], [3], [8]–[10].

Imaging modalities such as ultrasonography, small bowel barium study and multidetector-row computed tomography (MDCT) have been used to diagnose SBO, to identify possible causes and to rule out other intra-abdominal conditions that cause similar symptoms [11]. There are several published series describing the value of MDCT features in decision-making regarding surgery in patients with adhesion-related SBO [3], [4], [10]. However, validating these imaging predictors in combination to distinguish SBO patients who require surgery from those who can be clinically observed is still an important clinical issue. Thus, the purpose of our study was to evaluate retrospectively all MDCT features for their value in predicting disease progression in patients with adhesion-related SBO, and more specifically, to weight these MDCT imaging parameters in predicting the requirement for surgery.

## Materials and Methods

### Patient collection and inclusion criteria

This retrospective study was conducted at a tertiary referral medical centre from January 2006 to December 2011. The institutional Review Board for Human Investigation (TSGHIRB 099-05-027) approved this study, and the requirement for written informed consent was waived because patient anonymity was strictly maintained and the study was an observational one. In a search of the institutional database using the International Classification of Diseases, 9^th^ Revision code 560.81, we found 176 patients with a final diagnosis of adhesion-related SBO that were hospitalized during the period covered by our study. Of these patients, we excluded 25 because they had not received an MDCT scan (n = 17) or were clinically observed in our hospital for less than 5 days (n = 8).

Our final study population consisted of 151 patients (men  =  89 and women  =  62; mean age, 62.3 years). These patients were divided into two groups. The surgery group consisted of those in whom surgical intervention was performed for this episode of SBO; and the observation group consisted of patients who did not undergo surgery and whose symptoms of obstruction were eventually relieved.

### Collection of the clinical data

One clinician, who did not participate as a reader, retrospectively reviewed the medical records of these 151 patients with adhesion-related SBO. He recorded data including age, sex, clinical symptoms, underlying disease, and laboratory parameters at admission, morbidity, mortality, and hospital stay for all patients. Each patient received abdominal MDCT during admission or in the emergency room. In our hospital setting, surgical intervention was decided based on the clinical presentations (such as fever, tachycardia, leucocytosis, persistent abdominal pain) and MDCT features. The need of operative exploration was judged by consensus of two consulting surgeons. In our surgical group, 39 patients were decided for operative exploration at the time of surgical consultation, and 24 patients were initially treated with conservative therapy and finally receive surgery. Surgical decision of these 24 patients was based on the persistent abdominal symptoms, peritonitis, or evidence of bowel ischaemia or perforation. Here are the following recorded reasons: (1) progress to generalized or localized peritonitis by symptoms (n = 7), (2) visceral perforation (n = 1), (3) evidence of bowel ischaemia by either clinical presentations or follow-up MDCT findings (n = 4), or (4) persistent abdominal symptoms of intestinal obstruction that did not resolve within 5 days after conservative therapy was initiated (n = 12) [5], [12]. Mortality was defined as 30-day or in-hospital death. Morbidities included sepsis, acute renal failure, acute respiratory failure, shock, hepatic failure, pleural effusion, upper gastrointestinal bleeding, and an episode of coronary artery disease during the same hospitalization.

### MDCT protocol and techniques

In our institution, computed tomographic scanning protocol for SBO were performed on a 64-MDCT scanner from the domes of the diaphragm to the symphysis pubis by using the following scanning parameters: slice thickness, 5 mm, collimation, 16 × 0.75 mm; table feed/rotation, 12 mm; slice width, 0.75 mm; beam pitch, 1.5; tube voltage set at 120 kVp; and the maximum tube current limited to 250 mA. Unenhanced MDCT scans were not routinely performed with oral administration of water or iodinated positive contrast material. MDCT with administration of non-ionic iodinated contrast material (iodine concentration, 350 mg/ml, Omnipaque, GE Healthcare, Norway) was obtained unless the patient was in a critical condition and could not tolerate this procedure. An on-duty radiologist made the decision about oral and intravenous administration depending on the patient’s general condition and tolerance. At the beginning of the contrast-enhanced CT scan, 80–100 mL of contrast material was injected intravenously through a 20-gauge cannula at 2–3 mL/s using an automated power injector. The delay between the start of contrast administration and the start of MDCT scanning was approximately 70 seconds for the portal venous phase. Image reconstructions in the coronal plane were routinely created at the scanner console by the technologist immediately after completion of the scan. The scanned data were retrospectively reconstructed using 1.25-mm thickness and 0.625-mm spacing. The coronal reformations were created using 2.5- to 3-mm collimation extending from the anterior abdominal wall to the spine. The 5-mm axial and the 2.5-mm coronal images were then transferred to the Picture Archiving and Communications System (PACS) (Agfa IMPAX 4.0; Richmond, VA) as separate series for interpretation.

### Interpretation of the MDCT findings in patients with SBO

The names and identifying patient record numbers were electronically removed from all images for each MDCT examination prior to loading of the cases on a workstation for review. Two gastrointestinal radiologists, with 7 and 11 years of experience respectively, were asked to review the MDCT appearances. The two radiologists individually reviewed the MDCT images. The radiologists were aware that these patients had adhesion-related SBO but they were blinded to the clinical results and surgical decision of each patient in the review. Discrepancies between the two reviewers were resolved by further consensus readings. For this assessment, we recorded the MDCT images for each patient as follows: (1) bowel wall thickness (cm), (2) bowel lumen diameter (cm), (3) presence of a transitional point, (4) degree of obstruction, (5) presence of the small bowel faeces sign, (6) air–fluid level, (7) presence of intraperitoneal fluid, (8) mesenteric fatty infiltration, (9) presence of a closed loop, and (10) presence of the whirl sign. In order to standardize the MDCT measurement, definition of these imaging parameters were list as follows.

#### Bowel wall thickness

The abnormally thicken bowel wall was determined with a cut-off value of 0.3 cm[13].

#### Bowel lumen diameter

The abnormally distended small bowel was determined with a cut-off value of 3 cm calculating from outer wall to outer wall.

#### Degree of obstruction (Fig. 1)

SBO can be defined as three types of obstruction [3], [13]. On MDCT, “low-grade” was defined if a moderate amount of gas and liquid stool were observed in the ascending colon, “high-grade” if only minimal gas or liquid stool was seen in the ascending colon, and as “complete” if the ascending colon was totally collapsed without gas or fluid in its lumen. The major difference between “high-grade” and “complete” SBO was that small amount of material still passing distal to the obstruction in “high-grade”. This phenomenon indicates that “high-grade” SBO is delayed but not totally occluded in passage of material or stasis.

**Figure 1:**
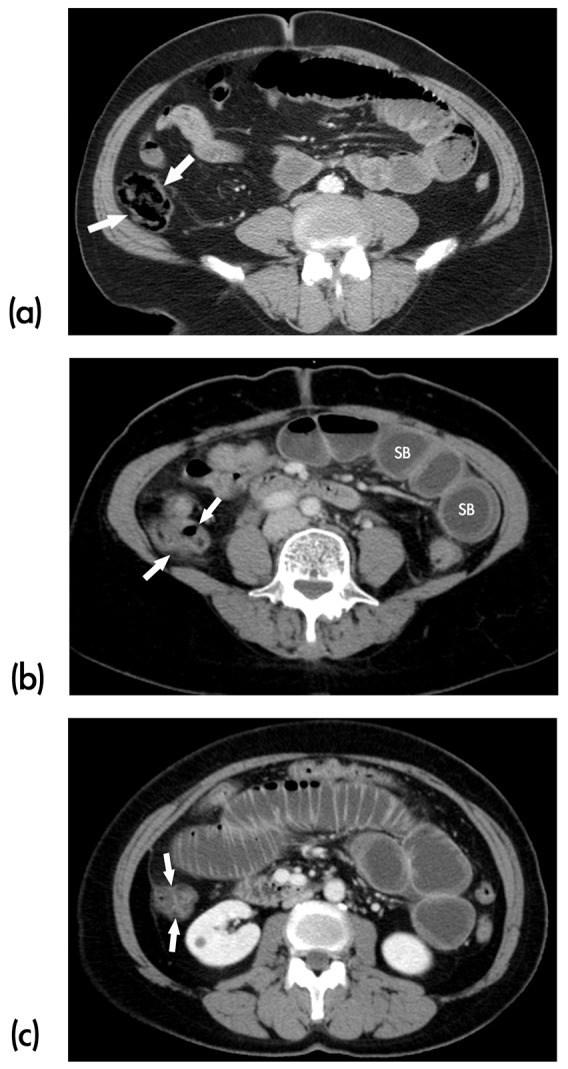
Three degrees of SBO defined by MDCT images. (a) “Low-grade partial” SBO. Note the moderate amount of gas and faeces in the ascending colon (arrows). (b) “ High-grade partial” SBO. Note the small amount of gas and fluid in the ascending colon (arrows). There is no evidence of small bowel wall thickening or ischemia. The maximal diameter of the obstructed fluid-filled small bowel (SB) is measured larger than 3.0 centimeter. (c) “Complete” SBO. Note the complete collapse of the ascending colon (arrows) with no appreciable gas or fluid.

#### Presence of transitional point

Bowel tracking in an antegrade or retrograde manner on the workstation of MDCT could help to accurately identify the transition point. It was determined by identifying a caliber change between dilated proximal and collapsed distal small bowel loops.

#### Presence of small bowel faeces sign (Fig. 2)

It was defined as presence of faeces-like material mixed with gas bubbles and fluid in the lumen of dilated small bowel loops proximal to the site of obstruction [14].

**Figure 2:**
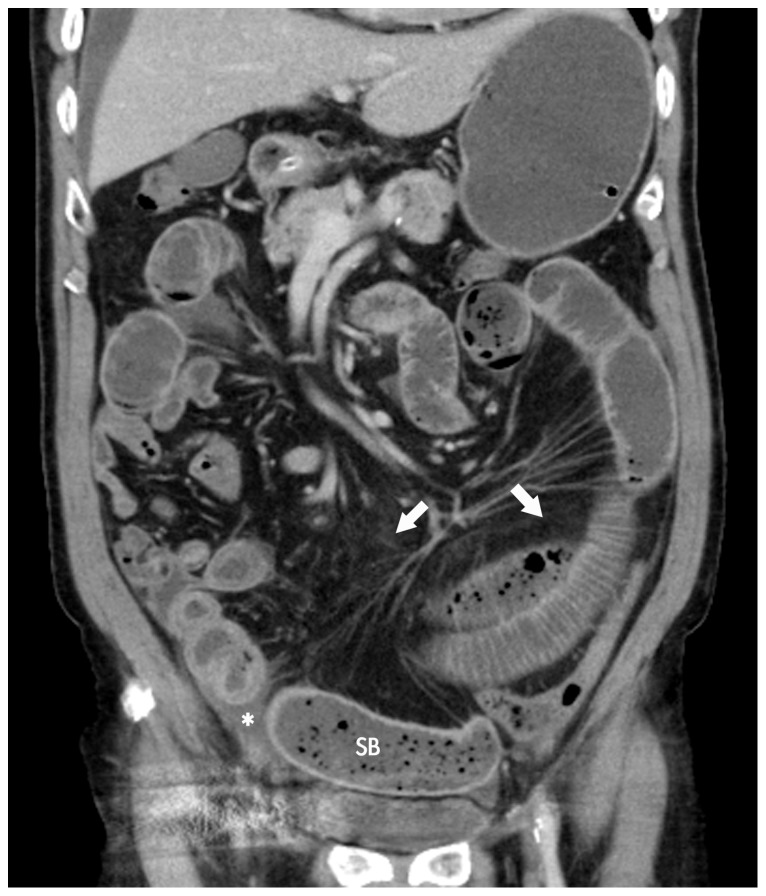
Small bowel faeces sign. Coronal MDCT image shows faeces-like material mixed with gas bubbles and fluid at the distal small bowel (SB). The finding is frequently seen proximal to the site of obstruction. Mesenteric fatty infiltration (arrows) and small amount of intraperitoneal fluid (asterisk) are also observed.

#### Presence of close loop (Fig. 3)

A closed loop obstruction is when there is isolated loop of dilated bowel with decompressed proximal and distal bowel loops. The proximal and distal transition points are located either at the same point or very close to each other. Based on the description, we defined as “a U- or C-shaped dilated small bowel loop with a radial configuration of stretched mesenteric vessels converging toward the site of torsion” [15] on MDCT.

**Figure 3:**
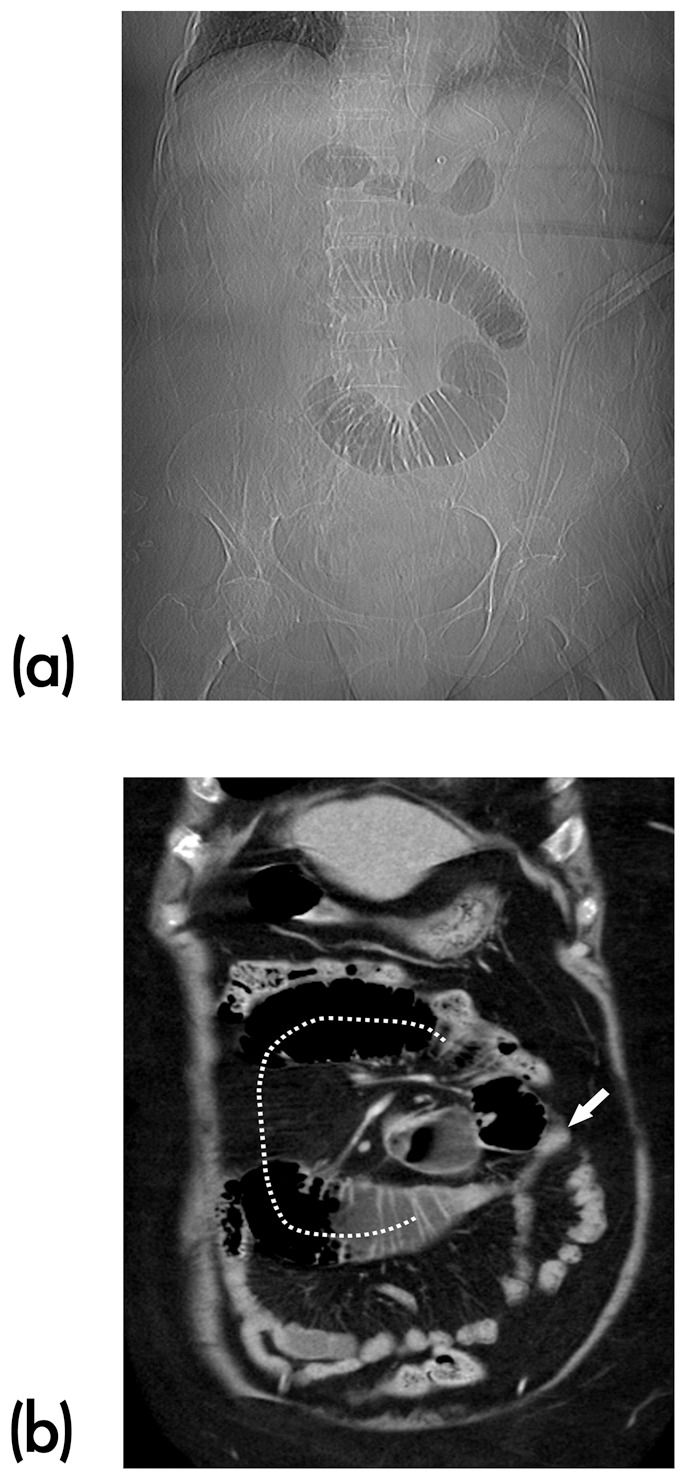
Closed-loop sign. (a) Abdominal radiograph shows a C-shaped configuration of the bowel loops in the center of the abdomen, a finding that indicates closed-loop obstruction. (b) On coronal MDCT image, the affected loops (dotted line) are filled with gas. The stretched mesenteric vessels converging toward the site of torsion (arrow).

#### Presence of whirl sign (Fig. 4)

A tightly twisted mesentery was defined as the “ whirl sign” [16].

**Figure 4:**
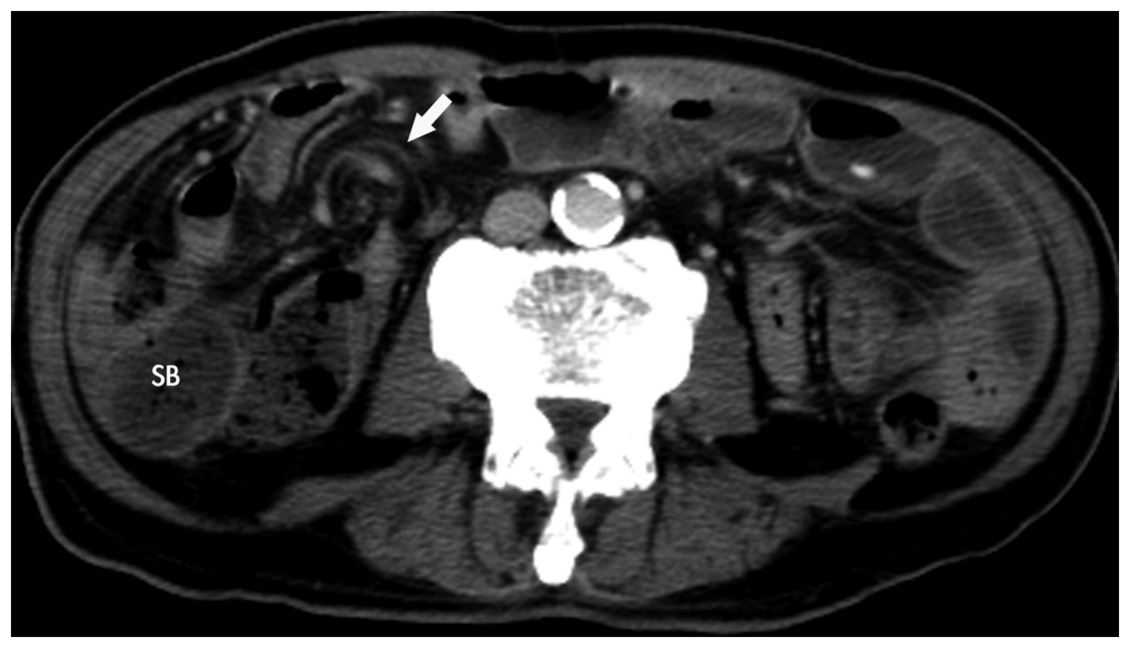
The “whirl” sign. Axial MDCT image shows the whirl appearance of twisted mesenteric vessels (arrow), supplying the obstructed small bowel (SB) lying laterally to the colon.

In addition, MDCT signs of small bowel ischaemia or perforation that complicated with adhesion-related SBO were also evaluated. The MDCT findings of bowel ischaemia or perforation included: (1) decreased bowel wall enhancement, (2) pneumatosis intestinalis, (3) portal venous gas, and (4) bowel wall perforation with pneumoperitoneum.

### Statistical analysis

Categorical clinical variables and each MDCT feature were compared between the observation and surgery groups of patients using the *χ* ^2^ test. Differences in age and laboratory data between groups were analysed using Student’s *t*-test to determine whether each variable differed significantly between the two groups. A multivariable stepwise logistic regression analysis was used to test the univariate models that demonstrated the best predictors of surgery *versus* observation. Using these data, the sensitivity, specificity and accuracy of the criteria for differentiating the surgery group from the observation group were evaluated. In order to obtain significant and optimal results and to efficiently estimate the error rate, we also performed a ten-fold testing cross-validation model to validate the estimation of predictor errors. The simulation model was designed by randomizing selection of our cases (80% to 90% of cases for each testing) in the two groups, and re-calculating the sensitivity, specificity and accuracy in each testing. A *p*-value of less than 0.05 was considered significant. All statistical analyses were performed using the SPSS software package (version 15.0; SPSS, Chicago, IL).

## Results

The study group included 151 patients comprising 89 men and 62 women aged 18–95 years (mean age, 62.3 years). We divided these patients into two groups: 63 (41.7%) patients underwent surgical exploration at the episode of obstruction (surgery group), and the remaining 88 (58.3%) patients received conservative management only during the admission (observation group). In observation group, improvement of SBO was decided by resolution of abdominal symptoms and no evidence of obstruction on follow-up images. In the surgery group, the interval between the onset of symptoms and surgery ranged from 8 to 432 hours (mean 69.6 hours). Among the conservatively managed patients, there was no evidence of bowel perforation or ischaemia during their hospitalization. The admission period of the patients in the observation group ranged from 5 days to 35 days, with an average of 8.4 days. Table 1 shows the demographics, clinical symptoms and laboratory data of these patients. The clinical symptoms, including fever (> 38°C), heart rate (> 100/min), systolic blood pressure (< 90 mmHg), vomiting, abdominal pain, muscle guarding and constipation, have been examined. Among these clinical symptoms, tachycardia (heart rate > 100/min) and muscle guarding showed statistical significances between surgery and observation groups. The laboratory parameters, including white blood count, C-reactive protein, blood urea nitrogen, creatinine, amylase, lipase, and arterial blood gas, were recorded. SBO patients showed significant higher levels of white blood count, C-reactive protein and lipase in surgery group than those in observation group. For the higher lipase level, it indicated that patients in the surgery group have a relatively higher risk with acute pancreatitis than those in the observation group. Acute pancreatitis caused by the obstructed bowel loops was sometimes evident by a dilated common bile duct where the surrounding inflammatory cells could potentially block the extrahepatic bile duct or elevate pressure in the biliary system. However in our study, we did not find the difference between the two groups (n = 6 in the surgery group, and 4 in the observation group. *p* = 0.32) in the MDCT finding of a dilated common biliary duct, indicating that the surgical decision was judged by high lipase level rather than a dilated common bile duct.

**Table 1:** Comparison of clinical and laboratory parameters in the surgery and observation groups.

Clinical and laboratory parameters	Surgery group (N = 63)		Observation group (N = 88)		*p* value*
Age (mean, range)	64.5	(31–90)	59.2	(18–95)	0.08
Sex					0.36
Male	41		48		
Female	22		40		
Clinical symptoms					
Fever (> 38°C)	17		28		0.32
Heart rate(> 100/min)	18		12		0.02*
SBP (< 90 mmHg)	24		23		0.11
Vomiting	28		42		0.41
Abdominal pain	53		81		0.10
Muscle guarding	14		9		0.04*
Constipation	8		10		0.49
Laboratory findings					
White blood count (×10^3^/µl)	10.8	(2.4∼29.0)	8.9	(1.4∼19.3)	0.02*
C-reactive protein (mg/l)	5.9	(0.08∼20.7)	2.6	(0.04∼12.5)	0.01*
Blood urea nitrogen (mg/dl)	25.5	(9.2∼41.3)	21.7	(5.2∼36.7)	0.18
Creatinine (mg/dl)	1.7	(1.4∼2.0)	2.0	(1.0∼3.1)	0.80
Amylase (mg/dl)	107.9	(24∼704)	107.7	(20∼700)	0.99
Lipase (mg/dl)	108.2	(18∼575)	30.7	(11∼168)	0.02*
pH value	7.42	(7.29∼7.61)	7.43	(7.34∼7.55)	0.75
Bicarbonate (mEq/l)	25.4	(13.3∼37.9)	24.5	(14.6∼30.1)	0.42

SBP, systolic blood pressure; pH, the hydrogen ion concentration.

Categorical data are expressed as number of patients, and continuous data are expressed as mean value (range of the data).

* *p* value < 0.05.

Table 2 summarizes the MDCT characteristics that distinguish SBO patients in the surgery *versus* observation groups. Univariate analysis showed several significant differences between the surgery and observation groups. The surgery group was significantly associated with high-grade or complete obstruction (*p* = 0.003), presence of mesenteric fatty stranding (*p* = 0.003), intraperitoneal fluid (*p* < 0.0001), and absence of the small bowel faeces sign (*p* = 0.022), whereas the observation group was significantly associated with low-grade obstruction, absence of mesenteric fatty stranding and intraperitoneal fluid, and presence of the small bowel faeces sign. The presence of intraperitoneal fluid was the most significant predictor in the surgery group (n = 45 in 63 patients, 71.4%) compared with the observation group (n = 32 in 88 patients, 36.4%), (*p* < 0.0001). No significant differences were found between groups for the frequency of bowel diameter less than 3 centimetres, bowel wall thickness greater than 2 millimetres, air–fluid levels, recognition of a transitional point, presence of a closed loop and presence of a whirl sign.

**Table 2:** Comparison of MDCT features in the surgery and observation groups.

MDCT features	Surgery group (N = 63)	Observation group (N = 88)	*p* value*
Bowel diameter (> 3 cm)	57	73	0.14
The degree of obstruction			0.003*
Low grade	19	51	
High grade	14	9	
Complete	30	28	
Small bowel faeces sign	19	42	0.022*
Mesenteric fatty stranding	47	45	0.003*
Maximal thickness of bowel wall (>2 mm)	9	15	0.41
Air-fluid level	59	84	0.38
Transitional point	53	70	0.31
Intraperitoneal fluid	45	32	<0.0001*
Close-loop sign	7	1	0.09
Whirl sign	1	5	0.2
Signs of bowel ischaemia and perforation			
Decreased bowel wall enhancement	5	2	0.1
Pneumatosis intestinalis	3	1	0.17
Portal venous gas	2	0	0.09
Bowel wall perforation with pneumoperitoneum	1	0	0.23

Categorical data are expressed as number of patients.

* p value < 0.05.

MDCT findings of bowel ischaemia or perforation that complicated with SBO are important criteria for surgical decision, although the data did not show significant results between the two groups (Table 2). In this study, we found that there was relatively small number of adhesion-related SBO patients having these MDCT features at the episode of obstruction. In observation group, only three patients with MDCT findings of decrease bowel wall enhancement (n = 2) and pneumatosis intestinalis (n = 1) were not recommended for surgery, because of the subsided clinical symptoms.

Multivariable stepwise logistic regression analysis showed that high-grade or complete obstruction, the absence of the small bowel faeces sign and the presence of mesenteric fatty stranding and intraperitoneal fluid were the most significant predictors of SBO that required surgery. We noticed that the sensitivity and specificity of intraperitoneal fluid are 71.4% and 63.6%, respectively, those of the high-grade or complete obstruction are 69.8% and 58.0%, those of mesenteric fatty stranding are 74.6% and 48.8%, and those of the absence of the small bowel faeces sign are 30.2% and 52.3%. Through a ten-fold testing cross-validation, the range of sensitivity, specificity and accuracy of each parameter are not significant (Table 3). When included together in a model, the highest odds ratio (OR) was achieved for the presence of intraperitoneal fluid (OR  =  4.38), followed by the presence of high-grade or complete obstruction (OR  =  3.19), mesenteric fatty stranding (OR  =  2.81) and the absence of the small bowel faeces sign (OR  =  2.11) (Table 3). When two or more of these four criteria were used in combination randomly, we could identify 50 to 57 of 63 patients (79.4%∼90.4%) in the surgery group and 53 to 72 of 88 patients (60.2%∼81.8%) in the observation group. When three or four of the four criteria were used randomly, we could identify 58 to 61 of 63 patients (92%∼96.8%) in the surgery group and 66 to 77 of 88 patients (75%∼87.5%) in the observation group. Finally, we achieved a high specificity of 98.6% and specificity of 90.9% (Table 4) by using all four MDCT criteria to determine those patients with adhesion-related SBO who required surgery.

**Table 3:** Sensitivity, specificity, accuracy values and odds ratio of MDCT features for predicting adhesion-related SBO requiring surgery.

	Odds ratio†	Sensitivity (% (number of patients)) (n = 63)	Specificity (% (number of patients)) (n = 88)	Accuracy (% (number of patients)) (n = 151)
Degree of obstruction	3.19 (1.61–6.33)	69.8% (67.9 ∼ 71.9)	58.0% (55.0 ∼ 60.8)	63.6% (63.2 ∼ 63.9)
Small bowel faeces sign	2.11 (1.07–4.18)	30.2% (24.5 ∼ 33.3)	52.3% (48.1 ∼ 57.5)	59.6% (48.4 ∼ 59.6)
Mesenteric fatty stranding	2.81 (1.39–5.68)	74.6% (71.4 ∼ 78.6)	48.0% (46.3 ∼ 51.9)	59.6% (58.1 ∼ 60.7)
Intraperitoneal fluid	4.38 (2.18–8.79)	71.4% (68.4 ∼ 75.0)	63.6% (62.0 ∼ 66.3)	66.9% (65.4 ∼ 69.3)

† Data in parenthesis is the 95% confidence interval in odds ratio, and data in parentheses are range of multi-fold cross-validation in sensitivity, specificity and accuracy. The final statistical model included only those predictor variables that were found to be statistically significant in the multivariable analysis.

**Table 4:** Number of patients who would be identified by using combinations of MDCT findings to predict adhesion-related SBO patients requiring surgery.

No. of MDCT findings†	Surgery group (n = 63)	Observation (n = 88)
2	50∼57 (79.4%∼90.4%)	53∼72 (60.2%∼81.8%)
3	58∼61 (92%∼96.8%)	66∼77 (75%∼87.5%)
4	62 (98.4%)	80 (90.9%)

† Predictions rely on one or more of the following findings: intraperitoneal fluid, degree of obstruction, mesentery fatty stranding and the absence of small bowel faeces sign. In surgery group, the data shows numbers of patients with positive findings were operated; and in observation, the data shows numbers of patients with negative findings were not operated.

## Discussion

SBO has a variety of possible pathological causes. The leading cause of SBO is adhesions, which account for 60% of cases, followed by malignancy, Crohn’s disease, and hernias [6], [9], [15]. In this study, we focused on SBO caused by adhesion, and the diagnosis and localization of SBO was based on a combination of clinical history, physical examination and radiological examinations. MDCT is currently believed to be the standard imaging modality for diagnosing the aetiology and predicting the severity of obstruction in these patients [5], [6], [12], [13]. Signs of strangulation, perforation or infarction should be detected to allow for more timely and appropriate surgical management.

In general, adhesion-related SBO usually begins when the normal luminal flow of intestinal contents is completely interrupted. The small intestine proximal to the obstruction becomes disproportionally dilated, and secretions are prevented from passing distally. As time progresses, patients develop nausea and vomiting, and bowel sounds become hypoactive or entirely absent. The wall of the obstructed bowel becomes more and more oedematous as the process continues, which leads to a transductive loss of fluid into the peritoneal cavity. If not diagnosed and properly treated, vascular compromise leads to bowel ischaemia and further morbidity and mortality [7], [10], [12], [15], [16]. Thus, we believe that determining the predictive value of MDCT features for surgical decision-making is essential for appropriate treatment and to prevent delay.

In our multivariable analysis, adhesion-related SBOs requiring surgery were significantly associated with the presence of intraperitoneal fluid, high-grade or complete obstruction, mesenteric fatty stranding and the absence of the small bowel faeces sign, whereas SBOs not requiring surgery were associated with the opposing signs. The presence of intraperitoneal fluid was the most useful predictor, a finding consistent with those of two previous studies [4], [10]. It has been suggested that when the obstructed small bowel loop begins to undergo an irreversible process of damage, this tends to result in a transductive loss of free fluid into the peritoneal cavity. Moreover, the most recent study by Zielinski et al [10] suggested an algorithm that included the clinical, laboratory and radiographic findings in combination to judge the need for surgical exploration. Our study only stressed on the MDCT features, but similar with Zielinski et al [10], we both agreed that MDCT is the modality of choice for preoperative evaluation, and the recognition of a transitional point on MDCT scan is usually not an indication for surgery.

Unlike the situation with blunt abdominal trauma [17], where the attenuation of intraperitoneal fluid may not be helpful in triaging a patient to observation or exploratory surgery, there is always an accumulation of low-density intraperitoneal fluid in adhesion-related SBO. Even though 32 of the patients (36.4%) in the observation group had free intraperitoneal fluid, it still a suspicious finding that should be closely follow up. If there is any clinical or radiological finding of strangulated bowel, repeat imaging or surgery may be required.

Several previous reports have mentioned the important role of identification of a transitional point in adhesion-related SBO [13], [14], [18]. Lujan et al stated that if a transitional point could be recognized preoperatively, a successful laparoscopic adhesiolysis of the band was much more likely to be achieved [18]. However, in this study, we did not find any difference between the surgery and observation groups in the frequency of this finding. Although multiplanar and three-dimensional MDCT capabilities can improve the detection of a transitional point [14], [19], identification of a transitional point may not be a significant MDCT feature for differentiating the need for surgery. Our study suggested that the transitional point could not be used as a reliable sign for assessing the severity of obstruction but could be used only to identify its aetiology of obstruction.

In our study, we found that the small bowel faeces sign was present more frequently in the observation group [(n = 19/63; 30.2%) *versus* (n = 42/88; 47.7%)]. A prior study reported that the presence of a faeces sign was more likely to occur in high-grade obstruction [14], thus having a tendency to associate with the need for surgical intervention. However, this conclusion is not a mainstream viewpoint. Many other studies have found that this sign is associated predominantly with low-grade sub-acute obstruction [13], [20], [21]. Our findings also support the possibility that presence of the small bowel faeces sign may be related to the chronicity of the obstructing process. It is believed that the more chronic the obstructing process, the more likely that it allows enough time for secretions to occur within the obstructed bowel loop and that it avoids complete obstruction.

Closed loop obstruction is a specific type of SBO in which two points along the course of a small bowel are obstructed at a single location. The closed loops appearance could be catastrophic if missing and should be considered as a surgical emergency. However, this sign did not show a statistical difference between the groups in our study. We have 8 cases of closed loop obstruction with one (as seen in Figure 3) being in the observation group. This patient who did not undergo surgery was because of the stable clinical condition not to recommend an emergent surgery. The symptom of obstruction was relieved by nonoperative medical management. Although only a relatively small number of patient populations, our result might not represent the general population of adhesive-related SBO. The closed loop sign can be initially observed, especially when not accompanying with the other critical MDCT signs for surgery.

Finally, bowel ischaemia and perforation are the two important pathological indications favouring a decision for surgery [7], [22]. Similar to previous studies [10], [23], our study also suggests that decreased bowel wall enhancement or poor flow of contrast material into a section of bowel indicate bowel ischaemia, whereas pneumatosis intestinalis, portal venous gas and pneumoperitoneum indicate necrosis and perforation. These findings, of course, should be important imaging criteria for surgical decision prospectively. However, with the advancement of knowledge, there is relatively small number of patients in adhesion-related SBO complicating with bowel ischaemia and/or perforation. The purpose of early surgical intervention in patients with SBO is to prevent the ongoing bowel ischaemia or perforation. Due to small number and data showed no statistical significance, we did not select these findings in combination to predict the need for surgery.

Treatment options for adhesion-related SBO include early surgery or conservative treatment [3]–[5], [10], [12], [13]. There is no consensus with regard to the best procedure to follow. On the basis of the results of our multivariable analysis, we established reliable imaging criteria for the diagnosis of SBO requiring surgery: when all four significant signs were present, a diagnosis could be made with a high sensitivity of 98.6% and specificity of 90.9%. Besides, we also could note that there was a significant improvement of prediction for surgery in our SBO patients when more than one of these four signs was detected. Although further prospective studies with a larger series of patients are required to determine whether these findings truly predict the need for surgery, we believe that our results are meaningful.

Our study has some limitations. First, this was a retrospective study and there was a small number of patients (n = 7) did not examine contrast-enhanced CT scan that potentially prohibit for accurately interpreting the MDCT imaging features. Second, the optimal time to perform surgery is not always a straightforward decision, and a few patients may hesitate to have surgery because of the surgical risk, even if the evidence of bowel loop strangulation is convincing. Third, we divided these patients into a surgery group and an observation group, as did Hwang et al [3]. Eight patients who presented with symptoms of partial SBO and were clinically observed for less than 5 days were not included in our study. In other previous reports comparing surgery and non-surgery groups, patients with a non-surgery period of less than 5 days were included in the observation group. The symptoms in these excluded patients may resolve spontaneously after their discharge from hospital, as they did in our observation group. These patients had been enrolled in the studies of Jones et al [4] or Zielinski et al [10], similar results have been observed. Because of the limited number and strict criteria of this study, we decided to exclude these patients.

In conclusion, the presence of intraperitoneal fluid, high-grade or complete obstruction, mesenteric fatty stranding and absence of the small bowel faeces sign were the most significant findings for predicting the requirement for surgery in patients with SBO caused by adhesion bands. The combined presence of these MDCT features is highly suggestive of the need for early surgical intervention and may therefore be helpful in providing a better outcome.
